# An early warning surveillance programme for detecting upper limb deterioration after treatment for breast cancer: A novel technology supported system

**DOI:** 10.1186/s12885-015-1636-8

**Published:** 2015-09-15

**Authors:** Delva Shamley, Karen Robb

**Affiliations:** 1 Clinical Research Centre, Faculty of Health Sciences, University of Cape Town, Anzio Rd, Observatory, 7925 Cape Town, South Africa; 2 Macmillan Cancer Care, Consequences of Cancer Treatment Collaborative, England, UK

## Abstract

Upper limb morbidity is a well-recognised consequence of treatment for breast cancer that can develop for up to 6 years after treatment. However, the capacity to fully integrate evidence-based rehabilitation pathways into routine care for all patients is questionable due to limited resources. A long term surveillance programme must therefore be accessible to all patients, should identify those at risk of developing morbidity and target the interventions at the high risk population of patients. The proposed model uses a surrogate marker for assessing risk of morbidity, incorporated into an Early Warning System (EWS), to produce a technology-lead, prospective surveillance programme.

## Correspondence

Early Warning Systems (EWS) are traditionally designed for the early detection of signs of acute critical illness and have been implemented in cardiac care[1] and intensive care units [2]. The system identifies patients at risk of developing complications and allows for the early intervention in order to prevent escalation into a fatal case. Key components of an EWS include: 1. Identification of risk factors; 2. Timely collection of information; 3. Decision making based on information and 4. Triggering of an intervention. We consider these components to be transferable to chronic conditions such as the adverse treatment-related effects seen in breast cancer survivors.

Most women diagnosed with breast cancer go on to have a normal life expectancy but those who develop pain after treatment for breast cancer experience diminished ability to carry out active daily living (ADL) tasks, reduced health-related Quality Of Life (QOL), and psychosocial distress [3]. Initiatives such as the National Cancer Survivorship Initiative in the UK have significantly raised the profile and awareness of consequences of treatment and the need for tailored interventions [4]. Consequences include lymphoedema (varied incidence, but usually develops within 3 years of initial treatment) and decreased shoulder mobility and pain (incidence of 30 % -50 %) [5]. Additional considerations are connective tissue changes such as scarring [6] and Axillary Web Syndrome [7], which are known contributory factors to arm morbidity. Our team has further enhanced our understanding of upper limb dysfunction by describing scapula deviations and altered muscle activity associated with patient reports of pain [8, 9]. Together with others [10] we have also shown that these complaints can occur for up to 6 years post-surgery, which suggests they may be latent effects of adjuvant therapies. Indeed, several studies have shown a strong association between observed movement deviations, pain and adjuvant therapies (chemotherapy and radiotherapy)[6–9].

However, not all patients develop pain and shoulder dysfunction under the same treatment conditions and in spite of the existence of evidence-based rehabilitation pathways defined for breast cancer [11], capacity to fully integrate these into routine care is questionable due to limited access to resources. Interventions must therefore target those at risk of developing upper limb morbidity.

## Identifying risk factors for the development of upper limb morbidity

### Shoulder pain and dysfunction

Systematic reviews and prognostic studies that have controlled for influential variables reduce risk factors to more invasive surgery, radiation therapy, high BMI (risk factor for Lymphoedema), severe acute postoperative pain and pre-operative anxiety [7, 12]. Attempts to identify clinical risk factors have been hindered by the complexity of the condition and the many clinical variables involved in cancer management.

Work from our team has shown that the Shoulder Pain and Disability Index (SPADI – a Patient Reported Outcome Measure) [13] identifies key functional limitations associated with high, intermediate and low levels of pain and is correlated with specific altered muscle activity and scapula deviation patterns. The SPADI therefore has the potential to be an accessible, simple surrogate marker for the early identification of patients at risk of developing advanced shoulder pain and dysfunction. Evaluation of the SPADI pain data in our study has shown that specific items could be scored highly and associated with observed movement deviations while others were not. Use of a mean or total score may thus have resulted in patients being missed. We therefore selected the 3 highest scoring pain items and scored these more highly in order to rate risk. Owing to the fact that high SPADI pain scores correlated to high SPADI disability scores, only pain is included in the algorithm [8, 9].

### Lymphoedema

Several risk factors have been associated with the development of lymphoedema but current opinion is that it is still not possible to predict who will develop this condition [14, 15]. However, evidence supports the use of early exercise interventions to reduce the incidence of lymphoedema in breast cancer patients [16]. An EWS would raise patient awareness and ensure a timely clinical response.

## Early detection of upper limb deterioration in breast cancer survivors

We are not aware of the existence of a risk-based early warning system (EWS) that tracks patients progress using a self-assessment and self-referral online system after treatment for breast cancer. A recent consensus for a similar prospective surveillance programme concluded that the absence of dedicated resources in most countries means we should be looking to include technology driven initiatives to address the issues of early detection and thus prevention [17].

## Proposed Early Warning System for breast cancer survivors

**Fig. 1:**
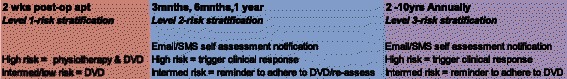
Summary of an Early Warning System to detect deterioration of the upper limb after treatment for breast cancer

The programme detailed below aims to provide a simple, yet effective system that can be seamlessly implemented into any clinical environment. It consists of defined points of assessment summarised in Fig. 1.

### Level 1-risk stratification: 2–3 weeks post-surgery

The first assessment occurs at the 2–3 weeks post-surgical visit where a brief risk-based questionnaire is utilised to triage patients. Implementation is achieved by the patient reading a concise description of the programme (either in writing or verbally by clinical staff depending on site resources) and then completing the questionnaire while waiting for her/his appointment. At the end of each clinic these are collected by either the breast care nurse or the physiotherapist and triaged according to the simple algorithm. High risk patients are then contacted by the physiotherapy department for an appointment within 2 weeks. This ensures the patient is able to achieve the arm position required for radiotherapy. Risk categories are described below and are based on evidence of levels of pain known to interfere with QoL and acitivties of daily life (ADL) and previous research (8,9). At this stage all patients’ hospital numbers and preferred contact details are entered onto the website for future surveillance. Where systems allow it, this information can be linked to clinical software in place.

All patients should be given a rehabilitation DVD or extensive booklet at discharge. The rehabilitation programme should contain education, advice, relaxation and an upper limb strengthening and mobility exercise regime (based on evidence of shoulder movement deviation). It should also provide education and advice on reducing the risk of developing lymphoedema.

### Level 2-risk stratification: 3 months, 6 months, 1 year post-surgery

All patients receive an email or SMS reminding them to access a website (hosted by the institution) in order to carry out an online self-assessment. This online assessment is similar to the one carried out at 2–3 weeks post –surgery but now includes questions related to the development of swelling/heaviness. On completion of the questionnaire, the responses are analysed and the patient is assigned to one of three categories and immediately receives one of the following three responses:

- Category 1. High risk – ‘Please contact’ ………. at this point the response is dependent on the institution and can include either; 1. a number for a breast care nurse or a physiotherapy department or 2. an email message is sent to the physiotherapy department. The self-referral system then triggers a clinical response and the patient is given a physiotherapy appointment.
- Category 2. Intermediate risk – ‘You may be at risk of developing shoulder problems. Please make sure you are adhering to the exercise programme on your DVD. Please re-do the assessment in 1 months’ time.’ (a reminder will be sent by an email or SMS). If the next assessment remains in this category they are referred to their local contact as per high risk category.
- Category 3. Low risk – ‘You are doing well. Please carry on with your exercise regime’.

### Risk stratification level 3: Annually from 2–10 years post-surgery

All patients receive an annual email or SMS reminding them to access the website and carry out a self-assessment. They are not given access to their previous ratings at this stage. The process from here is the same as in step 2 and involves a self-referral for the high risk category.

## Classification of risk

The following algorithm is proposed and forms the basis of the online analysis in order to allocate the patient to one of three risk categories.

1. SPADI pain items (Table 1):

**Table 1:** Five items included in the SPADI domain of Pain

1. At its worst?	0	1	2	3	4	5	6	7	8	9	10
2. When lying on the involved side?	0	1	2	3	4	5	6	7	8	9	10
3. Reaching for something on a high shelf?	0	1	2	3	4	5	6	7	8	9	10
4. Touching the back of your neck?	0	1	2	3	4	5	6	7	8	9	10
5. Pushing with the involved arm?	0	1	2	3	4	5	6	7	8	9	10

How severe is your pain?

Circle the number that best describes your pain where: 0 = no pain and 10 = the worst pain imaginable

  Q1 or Q3 score >5 = High OR 3 out of 5 questions score ≥5 = High
  3 of the 5 items score 3–5 = Intermediate
  all questions ≤ 3 = Low
2. Early signs of lymphoedema
  1. A heavy or achy feeling in your arm
  2. A tight sensation in your arm or hand
  3. Noticeable swelling in your arm, or hand
  4. Transient swelling. For example does your arm or hand suddenly swell for a short period of time and then go back to normal?
  5. Shirt sleeves that feel tight
  6. Ring or bracelet that starts to feel too tight
  7. Skin that “pits” or “dents” with finger pressure

If the patient responds with yes to any of the above questions they are asked to contact their breast care nurse or oncologist.

If each component of the surveillance programme is low on resource use, yet accessible and reliable, the likelihood of success is much greater. It should therefore be developed with all stakeholders to ensure commitment at each point but notably at the point of self-referral where a response from the health system is required.

The proposed surveillance programme offers a cost effective system to ensure that we reach as many patients as possible while working towards the inclusion of rehabilitation in the cancer management pathway [18].

## Institutional evaluation of the EWS

Two points of evaluation of the algorithm are suggested: 1. High risk patients presenting to the physiotherapy department (either from 2 week triage or from website referral) can be evaluated for appropriateness of referral. These patients can subsequently be followed up via their website responses and 2. The clinical algorithm can be evaluated for accuracy of identifying ‘at risk’ patients. This evaluation would require questionnaires to be sent to randomly selected patients in the intermediate/low risk categories to establish the presence over the last year of shoulder pain which had not been detected by the algorithm. The algorithm can then be adapted as required to meet the needs of the local population.
